# Supplementary material for: Ellagic Acid from Hull Blackberries: Extraction, Purification, and Potential Anticancer Activity
Source: Int J Mol Sci. 2023 Oct 16;24(20):15228. doi: 10.3390/ijms242015228 (PMC10607623; doi:10.3390/ijms242015228)
Supplement: Supplementary file 1 [file ijms-24-15228-s001.zip › ijms-2662833-supplementary.pdf]

Supplementary material

Ellagic Acid from Hull Blackberries: Extraction, Purification, and Potential Anticancer Activity

Jialuan Wang [1,†], Fengyi Zhao [1,†], Wenlong Wu [1], Lianfei Lyu [1], Weilin Li [2,*] and Chunhong Zhang [1,*]

[1] Jiangsu Key Laboratory for the Research and Utilization of Plant Resources, Institute of Botany, Jiangsu Province and Chinese Academy of Sciences (Nanjing Botanical Garden Mem. Sun Yat-Sen), Qian Hu Hou Cun No. 1, Nanjing 210014, China; wjl163youxiang@163.com (J.W.); zhaofengyi92@163.com (F.Z.); 1964wwl@163.com (W.W.); njbglq@163.com (L.L.)

[2] Co-Innovation Center for Sustainable Forestry in Southern China, College of Forestry, Nanjing Forestry University, 159 Longpan Road, Nanjing 210037, China

* Correspondence: wlli@njfu.edu.cn (W.L.); chzhang@cnbg.net (C.Z.)

† These authors contributed equally to this work.

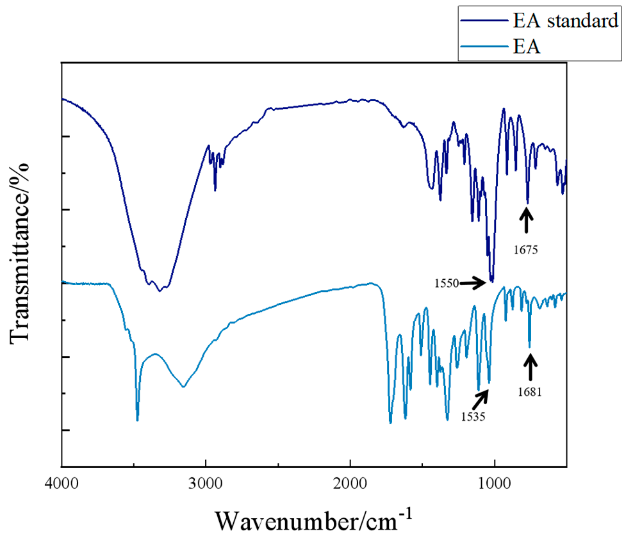

Figure S1. Infrared spectra. Dark blue is the infrared spectrum of EA standard; light blue is the infrared spectrum of experimentally EA extract.

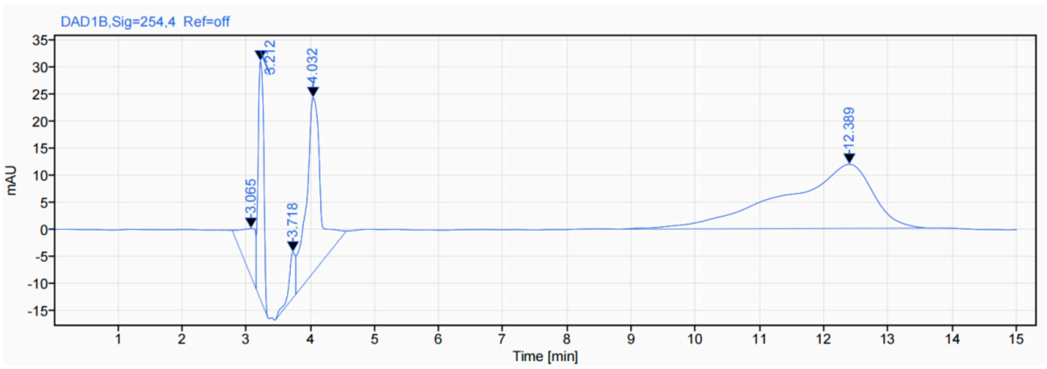

Figure S2. UPLC chromatogram.
